# Characteristic of Virulence and Parameters of Mixed Biofilm Formed by Carbapenem-Resistant *Pseudomonas aeruginosa* and *Proteus mirabilis* Strains Isolated from Infected Chronic Wounds

**DOI:** 10.3390/pathogens14060536

**Published:** 2025-05-27

**Authors:** Jana Wełna, Marta Napiórkowska-Mastalerz, Michał Cyrankiewicz, Tomasz Bogiel, Joanna Kwiecińska-Piróg

**Affiliations:** 1 Department of Microbiology, Faculty of Pharmacy, Collegium Medicum in Bydgoszcz, Nicolaus Copernicus University in Toruń, 85-067 Bydgoszcz, Poland; j.kwiecinska@cm.umk.pl; 2 Department of Biophysics, Faculty of Pharmacy, Collegium Medicum in Bydgoszcz, Nicolaus Copernicus University in Toruń, 85-067 Bydgoszcz, Poland; m.napiorkowska@cm.umk.pl (M.N.-M.); micy@cm.umk.pl (M.C.); 3 Department of Propaedeutics of Medicine and Infection Prevention, Faculty of Pharmacy, Collegium Medicum in Bydgoszcz, Nicolaus Copernicus University in Toruń, 85-067 Bydgoszcz, Poland

**Keywords:** biofilm, chronic wound, infection, mixed biofilm, *Proteus mirabilis*, *Pseudomonas aeruginosa*, virulence factors

## Abstract

A biofilm is a group of bacterial cells in the polysaccharide matrix bonded to the surface (biotic or abiotic). Clinicians now realize that most infections are biofilm-related. Biofilm infections are often induced by more than one bacterial species. The aim of this study is to characterize a mixed biofilm composed of *Pseudomonas aeruginosa* and *Proteus mirabilis* strains. Forty-six isolates derived from chronic wound infections were cultivated to establish mature biofilms. The biofilm biomass and cell viability were measured by colorimetric assays. *P. aeruginosa* strains were tested for the presence of virulence and biofilm-related genes. The quorum sensing assay using the biosensor strain was also performed. A mixed biofilm of *P. aeruginosa* and *P. mirabilis* was visualized using fluorescence microscopy. Four groups of *P. aeruginosa* and *P. mirabilis* pairs, also visualized with fluorescence microscopy, were distinguished based on the biofilm biomass growth and metabolic activity loss. The *exoY* gene observed among *P. aeruginosa* isolates was connected to the metabolic activity loss of the biofilm. Generally, the interactions between *P. aeruginosa* and *P. mirabilis* species are not uniform. It is crucial to further research the interactions between microorganisms in biofilms. This may provide information on the mechanisms of biofilm formation in the complicated chronic wound environment.

## 1. Introduction

A biofilm is an organized group of bacterial cells, encapsulated in the exopolysaccharide matrix and tightly bonded to the surface (biotic, such as tissue, and abiotic, such as biomaterial surface or polystyrene), which communicate and cooperate to survive. Biofilm development consists of a few maturation steps, mainly the reversible and irreversible attachment to the surface, microcolony formation, maturation, and dispersion [1]. A biofilm, through its structure, is also a protection barrier between bacterial cells and the environment. The polysaccharide matrix is a physical barrier hindering antibiotics at the site of infection. Extracellular DNA present in the matrix binds with antibiotic particles. Metabolically inactive cells may not respond to antibiotics, and lastly, a small percentage of antibiotic-resistant bacteria may reactivate the infection after antibiotic therapy. Bacterial cells encapsulated in biofilms manifest resistance, but the same strain in a planktonic form may be susceptible to antibiotics. This characteristic is defined as the antibiotic tolerance of biofilms. The strategy to break the biofilm structure and free bacterial cells before antibiotic therapy is one of many to be considered [2,3]. Another new definition linked to biofilms is antibiotic persistence. Antibiotic persistence is the outcome of the heterogeneity of bacterial communities within the biofilm. The most prevalent phenotype in the community manifests susceptibility, but a small percentage (about 1%) is resistant to the antibiotic and is capable of causing a recurrent infection [4,5]. The small resistant subpopulation, often with a slower metabolism, is made up of cells called persister cells. If the metabolism of persister cells is inhibited to the point where the cells cannot grow when placed on an agar plate, the cells are in a “viable but nonculturable” (VBNC) state [6]. Years of experience and research resulted in strict guidelines on how to use antibiotics. These guidelines are helpful to microbiologists and clinicians throughout the world [7,8]. Unfortunately, these guidelines are not applicable in the case of biofilm-associated infections. Their unique features forced scientists in the field to find new concepts and definitions [9].

Certain conditions are strongly correlated with biofilm prevalence at the site of infection. Cystic fibrosis patients are prone to *Pseudomonas aeruginosa* colonization in the lungs [10,11,12,13]. This prolonged colonization leads to biofilm formation and recurrent pneumonia [14,15]. Chronic otitis media is also a disease mostly caused by *P. aeruginosa* and linked to biofilm occurrence [16,17,18]. Chronic wounds, such as diabetic foot ulcers, venous leg ulcers, or pressure ulcers, are most likely to be colonized during the prolonged healing process, most often by *Staphylococcus aureus*, *P. aeruginosa*, and Enterobacterales [19,20,21]. Based on the literature, more than 80% of chronic wounds are colonized with biofilms [22].

Chronic wounds are characterized by stalling in the inflammatory phase [23]. The differences in the ongoing processes in the chronic and acute wound types resulted in the distinction of the ‘chronic inflammatory type’. In this inflammation process type, macrophages and fibroblasts behave differently due to the cells’ senescence [24]. Keratinocytes around the edge of the wound do not follow the correct migration process necessary for re-epithelialization. This abnormality leads to thickened wound edges [25]. The equilibrium between pro- and anti-inflammatory factors is disturbed in favor of pro-inflammatory cytokines [25]. The abundance of complex processes causes difficulties in chronic wound environment reconstruction in vitro. The interplay between the host’s immune system and complex wound microbiota is intricate, and the wound environment changes over time [25].

Based on the authors’ data, the most prevalent species in chronic wound infection are, respectively, *S. aureus* (46.1%), *P. aeruginosa* (35.0%), and *Proteus mirabilis* (10.6%). Amongst chronic wound infections, 75.1% carried a mixed infection with two or more pathogens (see Appendix A). The second most prevalent duo was *P. aeruginosa* and *P. mirabilis*. Based on these data, we chose to perform tests on these species, assuming that the prevalence of the mixed biofilm shows signs of cooperation between them.

The aim of this study is to characterize the mixed biofilm of *P. aeruginosa* and *P. mirabilis*, both isolated from chronic wound cases. A secondary aim is to investigate the influence of *P. mirabilis* on the formation of the *P. aeruginosa* biofilm and its overall condition.

## 2. Materials and Methods

### 2.1. Bacterial Strains

The bacterial strains for this research were selected from the collection of clinical isolates belonging to the Clinical Microbiology Laboratory of Antoni Jurasz University Hospital No. 1 in Bydgoszcz, Poland. The inclusion conditions were as follows: *P. mirabilis* or *P. aeruginosa* species, with isolated strains from chronic wound infections only, with an additional criterion for *P. aeruginosa* strain resistance to at least one of the carbapenems. Twenty-three strains of each species were selected. Before their use, the strains were frozen at −70 °C (Thermo Fisher Scientific, Waltham, MA, USA) in a Brain–Heart Infusion Broth (BHI, Oxoid, Altrincham, Cheshire, UK) with 10% glycerol (Avantor). Before the initiation of this study, the strains were stroked on Columbia Agar supplemented with 5% sheep blood (CAB, bioMérieux, Marcy-l’Étoile, France) and incubated at 37 °C for 24 h. A second passage of bacteria was used for further assays.

#### Strains Characteristics and Origin

The strains were derived from infected wound patients originating from 13 wards: Department of Geriatrics (*n* = 10), Department of Dermatology, Sexually Transmitted Diseases and Immunodermatology (*n* = 7), Chronic Wound Treatment Clinic (*n* = 6), Department of Vascular Surgery and Angiology (*n* = 5), Department of Nephrology, Hypertension and Internal Medicine (*n* = 4), Department of Anesthesiology and Intensive Care (*n* = 3), Department of Endocrinology and Diabetology (*n* = 3), Department of Cardiology (*n* = 3), Vascular Surgery Outpatient Clinic (*n* = 1), Department of Psychiatry (*n* = 1), Department of Forensic Medicine (*n* = 1), Department of Pediatrics, Hematology and Oncology (*n* = 1), and Department of Neurosurgery, Neurotraumatology and Pediatric Neurosurgery (*n* = 1).

The strains originated from chronic wounds, such as arterial ulcers (*n* = 5), ulcers caused by venous insufficiency (*n* = 1), diabetic foot ulcers (*n* = 5), pressure injuries (*n* = 9), and non-healing surgical wounds (*n* = 2). For one strain, it was not possible to determine the wound type.

The susceptibility of the tested strains was performed using the BD Phoenix™ automated identification and susceptibility testing system (Becton-Dickinson, Franklin Lakes, NJ, USA) and the Becton-Dickinson Phoenix™ panels NMIC-402. The susceptibility pattern of the strains is presented in Appendix B.

### 2.2. Biofilm Formation

The biofilm was prepared as follows: fresh overnight cultures on CAB were used to prepare a 0.5 MacFarland inoculum (approximately 1–2 × 10^8^ CFU/mL) in Phosphate Buffered Saline (PBS, BTL, Łódź Górna, Poland). Next, the initial inoculum was diluted 1:100 in Tryptic Soy Broth (TSB, Franklin Lakes, NJ, USA). Each well of the 96-well polystyrene plate (flat-bottom, sterile, NEST Biotechnology, Jiangsu, China) was filled with 100 µL of diluted inoculum for the single-species biofilm. For the mixed biofilm, strains were paired randomly. Each strain was added to each well in a volume of 100 µL to a total amount of 200 µL. Next, the wells were covered with sterile plastic lids and incubated at 37 ± 1 °C for 24 ± 1 h in a humid chamber to prevent evaporation. On the next day, the matured biofilm was gently washed three times with PBS to prepare it for the next steps. The investigation for each strain was performed in triplicate. A negative (sterile medium) control was included on each 96-well plate. The methodology was based on [26].

### 2.3. Crystal Violet Assay (CV Assay of Biofilm Biomass)

After the washing step, the plates were tapped vigorously to dispose of any planktonic cells and PBS residue. Then, the biofilm was fixed with methanol, following a 15-min incubation at 37 °C to become completely dry. Next, a 1% crystal violet (CV) solution (Avantor, Gliwice, Poland) in distilled water was added to each well at 200 µL. After 10 min, the plates were washed thoroughly with tap water and tapped vigorously again. Afterwards, the wells were filled with 300 µL of 96% methanol (Aura-Pol, Elbląg, Poland) and placed on a shaker for 15 min (100 rpm) to dissolve pigment crystals completely. Then, an absorbance was read at λ = 595 nm (Synergy, BioTek, Winooski, VT, USA.) and archived in the KC4 program version 3.4. (Synergy, BioTek, Winooski, VT, USA). Each strain was tested in triplicate. The methodology was based on [26].

### 2.4. Triphenyl Tetrazolium Chloride Assay (TTC Assay of Biofilm Metabolic Activity)

After the washing step, the remaining PBS and planktonic cells were carefully removed from the wells. Next, 180 µL of TSB and 20 µL of 0.1% triphenyl tetrazolium chloride (TTC; Sigma-Aldrich, St. Louis, MO, USA) in distilled sterilized water were added to each well and the plates were incubated at 37 °C for 8 h in a humid chamber. The wells were then emptied and washed with tap water to remove the TSB residue. Next, the wells were filled with 300 µL of 96% methanol and put on a shaker for 15 min to dissolve any pigment crystals completely. Absorbance was read at λ = 470 nm (Synergy, BioTek, Winooski, VT, USA) and archived in the KC4 program (BioTek, Winooski, VT, USA). Each strain was tested in triplicate. The methodology was based on [27].

### 2.5. Colony Forming Unit Assay (Quantitative Biofilm Cells Evaluation)

After the washing steps, all the wells were filled with 200 µL of PBS. The plates were sealed with a parafilm to avoid contamination and were sonicated using an ultrasonic bath (Clifton NE2-4D, Nickel-Electro Ltd., Oldmixon Crescent, Weston-super-Mare, UK). After the sonication procedure, the content of the wells was homogenized by mixing in a shaker for 15 min. Next, the wells’ content was diluted in PBS and plated on Tryptone-Soy Agar plates (TSA, Oxoid, Basingstoke, Hampshire, UK). After the 24 h incubation at 37 °C, the number of colony forming units (CFUs) was counted. Each strain was tested in triplicate. The methodology was based on [28].

### 2.6. PCR Assay (Biofilm Synthesis-Involved Genes) for Pseudomonas aeruginosa Strains

The PCR assay for selected *P. aeruginosa* genes, namely *toxA*, *lasB*, *exoT*, *plcH*, *exoY*, *exoU*, *aprA*, *algD*, *exoS*, *plC N*, *pilA*, and *pilB* genes, was performed as previously described [29,30].

For *lasR* and *rhlR* genes, the real-time PCR assay was performed on a Roche LightCycler II (Roche Diagnostics, Basel, Switzerland). The final reaction volume was 20 µL (1 µL of matrix DNA, 5 µL of molecular grade water, 5 µL of forward primer, 5 µL of reverse primer—both at the initial concentrations of 1 µM and 4 µL of EvaGreen MasterMix (Solis Biodyne, Tartu, Estonia)). The primers used were 3′-AGCAGCACGAGTTCTTCGAG-5′ (forward) and 3′-CTCTGCAGTGCGTAGTCCTT-5′ (reverse) for *lasR* and 3′-TCGGAAATGGTGGTCTGGAG-5′ (forward) and 3′-GGTCAGCAACTCGATCATGC-5′ (reverse). The amplification was carried out with the following thermal cycling conditions: 5 min at 95 °C for the initial incubation, 45 cycles of amplification consisting of 95 °C for 10 s, 61 °C for 20 s, and 72 °C for 20 s, followed by the high-resolution melting protocol (65 °C to 97 °C) to confirm the amplification specificity.

### 2.7. Quorum Sensing Assay

A quorum sensing assay was performed with the biosensor *Chromobacterium violaceum* 026 Tn5 strain. The strain was obtained from the National Collection of Type Cultures (United Kingdom Health Security Agency, Porton Down, Salisbury, UK). *C. violaceum* CV 026 (Tn5 mutant) responds to the C6-HSL by violacein production on agar plates [31]. The test was performed on the TSA plates. The tested strains and biosensor were stroked on the agar plate in close proximity. Next, the agar plates were incubated at 23 °C for two days. *P. aeruginosa* strains positive for acyl homoserine lactone production were indicated by the dark blue color of violacein produced by *C. violaceum*. The investigation for each strain was performed in duplicate.

### 2.8. Fluorescence Live Cell Imaging

For the imaging of the *P. aeruginosa* and *P. mirabilis* mixed biofilm, equal suspension volumes (1 mL) of each species at a 0.5 McFarland inoculum diluted 1:100 was added to the sterile 35 mm culture dish with a 20 mm glass bottom. The mixed culture was incubated for 18 h at 37 °C, washed three times with PBS afterwards, and stained with the LIVE/DEAD BacLight Bacterial Viability Kit (Invitrogen, Waltham, MA, USA) according to the manufacturer`s instructions. The investigation for each strain was performed in duplicate.

The microscopic imaging of bacterial biofilms was performed using a modified Elyra PS.1 system (Carl Zeiss Microscopy GmbH, Jena, Germany). The system is built based on a Zeiss Axio Observer Z1 inverted microscope coupled with a PCO.edge 4.2 sCMOS camera (PCO AG, Kelheim, Germany) and utilizes the structured illumination microscopy (SIM) technique to achieve an approximately two-fold enhanced resolution beyond the diffraction limit of conventional optical microscopy. All imaging experiments were conducted using the Zeiss plan-apochromat 20 × 0.8 M27 objective (Carl Zeiss Microscopy GmbH, Jena, Germany) in order to obtain a large field of view and considerable image detail at the same time. Biofilm samples were imaged using the SIM mode, with 488 nm laser lines for fluorophore excitation. Images were acquired sequentially in two emission channels: BP 495–590 + LP 750 and LP 655, for live and dead cells, respectively. Structured illumination was achieved by projecting patterned light onto the visualized objects, using 5 grid rotations and 5 phase shifts for each sample. The exposure time was set to 100 ms. Raw data from structured illumination were processed using Zeiss Zen Black 2.3 software, which applied SIM reconstruction algorithms to maximize clarity and minimize artefacts in the resulting SIM images. Then, due to the fact that the transmission functions of the filters used partially overlap, a principal component analysis (PCA) was used to separate the signal from live and dead cells. The contributions of the PC1 and PC2 components were further rescaled and assigned to the intensity of the green and red color components in the final images, respectively. The calculations were performed in the Matlab 2022b environment.

## 3. Results

### 3.1. Single Biofilm Versus Mixed Biofilm

**Figure 1:**
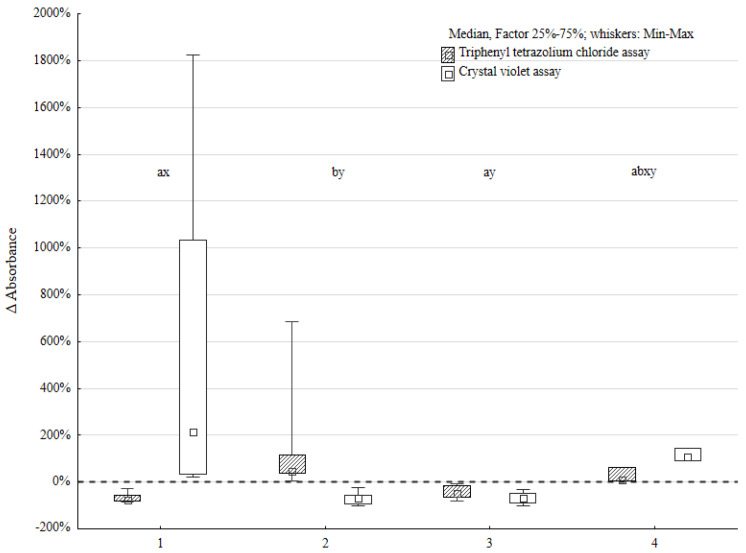
Metabolic activity of biofilms (TTC) and biofilm biomass (CV) changes expressed as Δ absorbance in four distinct groups of *Pseudomonas aeruginosa* (*n* = 23) and *Proteus mirabilis* (*n* = 23) mixed biofilms; the letters reflect statistically significant differences for TTC assay results (a, b) and CV assay investigation (x, y), respectively, to the observed groups: group 1—TTC ↓, CV ↑ (*n* = 5), group 2—TTC ↑, CV ↓ (*n* = 5), group 3—TTC ↓, CV ↓ (*n* = 11), and group 4—TTC ↑, CV ↑ (*n* = 2).

Four groups of strain pairs were determined based on the triphenyl tetrazolium chloride (TTC) and crystal violet (CV) methodology results. Five strain pairs manifested lower TTC and higher CV assay results (TTC ↓, CV ↑) compared to the sum of the *P. aeruginosa* and *P. mirabilis* single biofilm (serving as control). Five strain pairs manifested higher TTC and lower CV assay results (TTC ↑, CV ↓). The most numerous group, containing 11 strain pairs, obtained lower TTC and CV level (TTC ↓, CV ↓) results compared to the control. Two strains obtained higher TTC and CV results (TTC ↑, CV ↑) compared to the control (all the data are presented in Figure 1).

The tendency in interactions between *P. aeruginosa* and *P. mirabilis* was easily observed. The Wilcoxon signed-rank test proved the statistically significant difference between the results of the control group and mixed biofilm. This was positive for both the TTC and CV assays. However, the analysis performed using the χ^2^ test, to define the tendency of these interactions, showed no statistically significant difference.

The most distinct differences between the mixed biofilm and control were obtained in the group with lower TTC-based and higher CV-based assay scores. The difference in biofilm biomass compared to the control was up to 20 times greater in the mixed biofilm environment in these cases. This indicates that the interactions between *P. aeruginosa* and *P. mirabilis* species increased biofilm biomass production and slightly suppressed cell activity. It is possible that these strain pairs cooperated in biofilm formation.

The next group of bacterial pairs was characterized by a decrease in biomass production and an increase in the metabolic activity of bacterial cells. This may indicate that these pairs of strains competed for available components. They did not produce as developed of a biofilm as the control group.

The third group was characterized by a decrease in biofilm biomass and bacterial activity. This may indicate competitive interactions between strain pairs in this experiment.

In the latter group, the biofilm biomass was higher than in the control group and the metabolic activity of bacterial cells was increased. The increase in biofilm biomass was about two times greater than that in the control group. This may indicate the development of biofilm, although other processes that slow down biofilm production are probably also occurring at the same time. These processes may be competitive or mutual.

The statistical significance of the Kruskal–Wallis test results was confirmed by a post hoc analysis. The differences between the first and second groups were statistically significant in both tests (metabolic activity and biofilm biomass). However, there was no statistically significant difference in the metabolic activity of biofilm cells and biofilm biomass between groups 3 and 4.

### 3.2. Fluorescence Imaging of Mixed Biofilm

**Figure 2:**
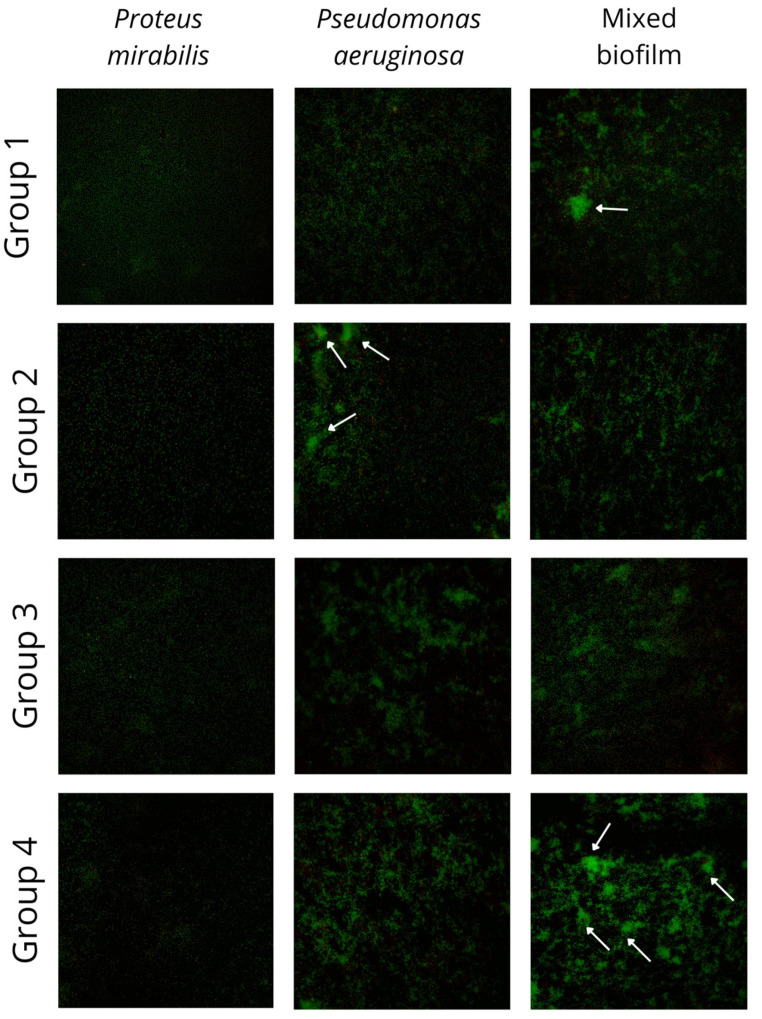
Pictures of mixed and single species biofilms of *Pseudomonas aeruginosa* and *Proteus mirabilis*. Biofilm was cultivated on sterile culture dishes and imaged using Elyra PS.1 (Carl Zeiss Microscopy GmbH) fluorescence microscopy system. Samples were imaged using super-resolution SIM mode with 488 nm laser lines for fluorophore excitation. A plan-apochromat 20×/0.8 objective was used. The visible area was about 0.25 mm × 0.25 mm. Images were acquired in the emission channel for live cells (marked by green fluorescent SYTO 9). Groups were divided based on the biofilm biomass (CV assay) and metabolic activity (TTC assay), respectively, to the observed groups: group 1—TTC ↓, CV ↑ (*n* = 5), group 2—TTC ↑, CV ↓ (*n* = 5), group 3—TTC ↓, CV ↓ (*n* = 11), and group 4—TTC ↑, CV ↑ (*n* = 2); white arrows indicate the microcolonies.

All 23 pairs of *P. aeruginosa* and *P. mirabilis* mixed and single biofilms were cultured and imaged using a fluorescence microscope. The results of the imaging are presented in Figure 2. The green fluorescence stain binds with bacterial nucleic acids. The distinctive differences between the four groups of mixed biofilms described before are visible in Figure 2. The differences between biofilm in groups 1 and 4 (biofilm biomass gain in mixed culture) and groups 2 and 3 (biofilm biomass loss in mixed culture) are visible in the pictures. The intensification of a green stain in groups 1 and 4, resulting from a higher number of bacterial cells in clusters, indicates enhanced biofilm production.

### 3.3. Pseudomonas aeruginosa Biofilm Impacted by Proteus mirabilis

Out of 23 mixed biofilm environments, in 16 pairs, the loss of CFU/mL was observed for *P. aeruginosa*. In 10 strains, the loss was near 1 log or 100%. In another six pairs, it was between 74% and 7% of the control CFU/mL loss. There was no difference between the control and mixed biofilm CFU/mL for two strains. Five *P. aeruginosa* strains exhibited a CFU/mL increase when incubated with *P. mirabilis*. For one strain, the increase was slight (9% of control CFU/mL), but for four strains, the difference was 77%, 163%, 389%, and 736%, respectively. For the two last strains, the difference is particularly significant.

**Figure 3:**
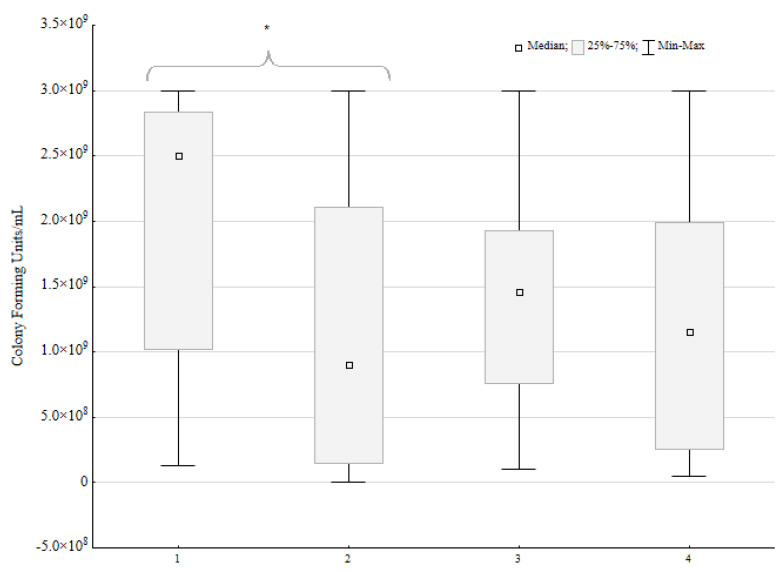
Number of colony forming units per mL in mixed biofilms compared to single biofilms (control) of *Pseudomonas aeruginosa* (*n* = 23) and *Proteus mirabilis* (*n* = 23). A sign test and Wilcoxon ranked test were performed; the statistically significant difference is marked with an asterisk.

The Wilcoxon ranked test was used to ensure that the *P. aeruginosa* CFU/mL loss under the influence of *P. mirabilis* was statistically significant (Figure 3). Both tests proved the significant impact of the *P. mirabilis* strains on *P. aeruginosa* CFU/mL (*p* < 0.05, α = 0.05). However, for the *P. mirabilis* species, there was no significant difference in the CFU/mL under the influence of *P. aeruginosa*.

### 3.4. Pseudomonas aeruginosa Toxins-Coding Genes Prevalence

The appearance of exotoxin-coding genes, which belong to type three secretion system effectors (except for the *toxA* gene), was high amongst *P. aeruginosa* strains. All the tested strains were positive for *exoT* and *toxA* genes; the presence of *exoY* and *exoU* genes reached 87.0%. For the *exoS* gene, the number of positive strains was relatively low, with eight strains exhibiting the presence of this gene, which was present among 34.8% of all tested strains only.

Only 52.2% of strains carried the *algD* gene, which encodes the enzyme involved in the alginate biosynthesis pathway. Even fewer strains carry the *pilA* and *pilB* genes for pilins A and B. Eight strains (34.8%) were positive for the *pilA* gene. Two of them carry the *pilB* gene simultaneously. No statistically significant differences were confirmed after performing the McNemar χ^2^ test.

The *lasB* gene, responsible for elastase (metalloprotease) production, was present in all the tested *P. aeruginosa* isolates included in the research.

The phospholipase C, encoded by the *plC H* (hemolytic phospholipase C) and *plC N* (non-hemolytic phospholipase) genes in *P. aeruginosa* strains, were noted among all and 41.7% of strains, respectively. No correlation between biofilm biomass and *plC N* carriage was confirmed.

### 3.5. Pseudomonas aeruginosa Homoserine Lactone Production

Of the 23 *P. aeruginosa* strains, 10 were positive for the production of the quorum sensing molecule N-hexanoyl-L-Homoserine lactone (C6-HSL), one of many bacterial autoinducers.

### 3.6. The Relation Between Biofilm Characteristics, Presence of P. aeruginosa Virulence Genes, and Production of C6-HSL

Based on the obtained results, Yates’s χ^2^ tests were performed to check the possible relation between each characterized virulence factor, the production of C6-HSL, and biofilm characteristics. The results of this analysis are presented in Table 1 and Table 2. The virulence factors present in all the tested strains were excluded from the analysis (*toxA*, *lasB*, *exoT*, *plC H*, *rhlR* genes).

The only statistically significant difference was manifested for the *exoY* gene and the differences in triphenyl tetrazolium salt absorbance in the single vs. mixed biofilm. The statistical difference was also observed between C6-HSL production and the presence of the pilin A-encoding *pilA* gene.

## 4. Discussion

The literature on mixed biofilms composed of different species of bacteria and fungi is extensive. However, there is a limitation in the meta-analysis of data, considering the different species and numerous methods used to characterize mixed biofilm. With the “mixed” [Title] AND “biofilm” [Title] search on Pubmed, 181 results have been documented since 1984. The number of articles published on mixed biofilms has, however, continued to increase over the last few years. This points out the presumption that interactions between microorganisms certainly take place. The following question emerged: what is the nature of these interactions, and what impact do they have on the infection site?

So far, the best method to assess the presence of biofilms in chronic wounds is fluorescence microscopy. This technique uses specific probes, enabling the exposition of bacterial cells, the biofilm matrix, and host cells in the environment. However, this method is expensive and has low throughput, making it highly impractical in screening [32]. Gram stain microscopy is useful in biofilm infection diagnosis according to ESCMID guidelines [32]. Although the Gram stain slide is crucial in microbiology diagnostics, it is hardly ever used to verify the presence of biofilms in materials. The difficulty in the recognition of biofilms in the smear may be the reason. The available literature is based on tissue biopsy microscopy to maintain the structure of the wound and biofilm [33,34].

After the identification of a biofilm in a chronic wound, there were attempts to characterize the interactions between different species of bacteria and fungi within the biofilm structure. However, these attempts are based on the characterization of the biofilm of reference strains or two pairs of reference strains versus two clinical isolates [35,36,37,38].

The method used in this paper is based on an in vitro biofilm cultivation in 96-well polystyrene plates. This method is far from the actual wound environment, excluding the presence of the host’s tissues and immune system. However, the interactions between microorganisms are complicated; thus, a simplified model was chosen.

Although there have been attempts to characterize interactions in dual-species biofilms [39,40,41], these works usually do not focus on the mixed biofilm of Gram-negative rods. The strong point of this research is the number of strains examined. Forty-six strains selected from chronic wounds were included in the experiments.

The only available research on the dual-species biofilm of *P. aeruginosa* and *P. mirabilis* in catheter-associated urinary tract infections is by Li et al. [42]. Li et al. emphasize the role of urease synthesized by *P. mirabilis* in the dual-species biofilm. Urease-producing strains were more likely to outgrow *P. aeruginosa* in the mixed biofilm, while strains incapable of urease production, as well as strains growing in the buffered artificial urine environment, were overgrown with *P. aeruginosa*. Thus far, it is not known if *P. mirabilis* produces urease in the wound environment. However, urea, which enhances urease production, is present in the stratum corneum [43]. Urea is essential for hydration, keratinocyte gene regulation, and enhances antimicrobial peptide synthesis [43]. The impact of urease production by *P. mirabilis* in the wound environment is unknown. However, it is clear from our in vitro experiments that for a majority of *P. aeruginosa* strains, cell growth was inhibited in the presence of *P. mirabilis*. The number of CFU per mL was lowered by 1.6 log for *P. aeruginosa* (*p* < 0.05) and 0.3 log for *P. mirabilis* (*p* > 0.05).

On the other hand, it has been shown that the biofilm biomass production between these two bacteria species may be strain-dependent. We were able to divide the *P. aeruginosa-P. mirabilis* pairs into four groups, depending on the characteristics of the formed biofilm. These results were supported with fluorescence imaging. This also applies to other experiments where the biofilm biomass was lower [44,45,46], comparable [39], or higher [46] than the sum of the biomass of monoculture biofilms. The difference in biofilm biomass production may result from the different organisms cooperating or competing with *P. aeruginosa*. However, the aforementioned studies tested only a single strain of each species, while our research compares 23 pairs of strains. This indicates that the results obtained may be strain-dependent.

The bacterial cell activity may also be strain-dependent. We divided strains into four groups, depending on the activity changes and biofilm biomass production in the monoculture and mixed biofilm. Similar results were obtained [47,48,49,50], where different pairs of fungi (*Candida* spp., *Rhodotorula* spp., *Trichosporon* spp.) strains were shown to have lower or comparable metabolic activity in mixed and monoculture biofilms.

All the tested *P. aeruginosa* strains were positive for the *toxA* and *exoT* genes, and 87.5% of strains had the potential to produce *exoU* and *exoY* toxins. This possibility increases strains’ virulence and the chance of infection. The type three secretion system exotoxins are responsible for cytoskeletal rearrangements of mammalian cells, the disruption of adhesion, and phagocytosis processes, while toxin A promotes tissue necrosis. All these factors can potentially affect the chronic wound healing process. Taking into consideration that all the tested strains are, at the same time, resistant to at least one carbapenem (see Appendix A), this indicates their very high virulence. This is also a problem reported worldwide for hospital-acquired *P. aeruginosa* strains [51]. Moreover, our analysis indicates the connection between the presence of the *exoY* gene in *P. aeruginosa* and the lower biofilm metabolic activity in mixed cultures with *P. mirabilis*. Lower metabolic activity may be connected to biofilm formation, while its presence in combination with the occurrence of secretion III system genes (*exoS, exoT, exoU,* and *exoY*) were linked by another research group previously [52].

Only 54.2% of strains carry the *algD* gene, which encodes enzymes involved in the alginate biosynthesis pathway. Even fewer strains carry the *pilA* and *pilB* gene encoding pilins. Nine strains (37.5%) are positive for the *pilA* gene, and two of them carry the *pilB* gene simultaneously. Alginate is a key component in the *P. aeruginosa* biofilm matrix, and bacterial pilins are necessary for bacterial adhesion and further biofilm formation [29]. These genes encode proteins known to participate in bacterial cell adhesion and other stages of biofilm formation. However, there was no significant correlation between gene carrying and biofilm biomass production.

The *LasB* gene is responsible for elastase (metalloprotease) production. Elastase is responsible for collagen and elastin degradation, it inactivates human immunoglobulin G, the serum α_1_-proteinase inhibitor, and complement system components [53]. The *P. aeruginosa* phospholipases C (hemolytic and non-hemolytic) are also an important factors in the development of infections. Phospholipases hydrolyze phosphatidylcholine and other phospholipids present in eukaryotic cells. Their role was closely determined in lung infections [54] but not chronic wound infections. For non-hemolytic phospholipase C, there is evidence that this enzyme participates in *P. aeruginosa* biofilm formation [55]. However, there was no correlation between *plC N* gene carrying and biofilm biomass production for the tested strains. This may result from the relatively small number of strains belonging to this particular group. Additionally, phospholipase C may be another virulence factor for chronic wound infection. The disruption of eucaryotic cells is a factor for further pathophysiological changes in the wound environment, promoting inflammation and a release of free radicals.

Quorum sensing is a complex system that differs between bacterial species. For *P. aeruginosa*, it helps to accommodate environmental cues. Quorum sensing for *P. aeruginosa* consists of three main autoinducers: N-butanoyl-homoserine lactone, N-Lauroyl-L-homoserine lactone, and quinolones. It is not excluded, however, that certain strains may produce other autoinducers. This was proved with the biosensor *C. violaceum* 026 Tn5 strain test, indicating the production of N-heksanoyl-homoserine lactone in 10 *P. aeruginosa* strains. Although the quorum sensing system for *P. aeruginosa* is mostly well-examined, the exact role of N-heksanoyl-homoserine lactone in this community organization is uncertain [56,57].

## 5. Conclusions

Mixed biofilm characterization is a complicated task. For now, the available methods include fluorescence microscopy, confocal microscopy, fluorescence in situ hybridization, or colony forming units assays. The scarcity of available information hinders new branch development. Thus, each study is valuable and may contribute to a better understanding of the problem.

The attempts to characterize mixed biofilms of *P. aeruginosa* and *P. mirabilis* are rare, despite the emerging problem in clinical experience. *P. aeruginosa* is a well-known virulent bacteria, while *P. mirabilis* is not a well-studied counterpart. Many aspects, such as quorum sensing systems, are not yet understood for *P. mirabilis*.

As shown in this article, similar to Li et al.’s [42] research results, *P. mirabilis* can generally hinder *P. aeruginosa* development. Interactions between two species may be strain-specific and could lead either to the general underdevelopment of biofilms or its advanced development. When only one pair of strains is the object of study and reference strains are used, the research results may be distorted because strains isolated from chronic infections may behave differently.

The fact that only the presence of the *P. aeruginosa* gene was tested is a disadvantage of this paper, because the gene expression levels may be more informative for the gathered data. This is the research direction that the authors plan to take in the future.

*P. aeruginosa* virulence factors may facilitate or hinder its biofilm growth in a mixed culture. The presence of the *exoY* gene in *P. aeruginosa* strains was connected to the lower biofilm metabolic activity in mixed cultures with *P. mirabilis*.

Although many genes are known to take part in biofilm formation in *P. aeruginosa*, their absence does not mean that the strains are impaired. Only 54.2% of strains carry the *algD* gene coding alginate, the main component of the *P. aeruginosa* biofilm matrix; however, all tested strains were able to form biofilms. This highlights the flexibility of the *P. aeruginosa* genome.

## Figures and Tables

**Table 1:** The Yates’s χ^2^ analysis of virulence factors and biofilm characteristics.

Virulence Factor/Gene	ΔCFU/mL *PAE*	ΔCV	ΔTTC
*exoY gene*	0.3348	0.9069	0.0050 *
*exoU gene*	0.0861	0.9069	0.1436
*exoS gene*	0.3627	0.1722	0.1364
*aprA gene*	0.3939	0.8978	0.8978
*algD gene*	0.4085	0.7524	0.5541
*plC N gene*	0.5257	0.4925	0.4925
*pilA gene*	0.9309	0.1364	0.5907
*pilB gene*	0.3793	0.5291	0.3276
*Quorum sensing*	0.7078	0.0737	0.0737

ΔCFU/mL *PAE*—the difference between the number of colony forming units of *Pseudomonas aeruginosa* per mL in single vs. mixed biofilm; ΔCV—the difference in crystal violet absorbance for single vs. mixed biofilm; ΔTTC—the difference in triphenyl tetrazolium salt absorbance in single vs. mixed biofilm; * indicates statistical significance.

**Table 2:** Yates’s χ^2^ analysis of virulence factors genes versus C6-HSL production of *Pseudomonas aeruginosa* (*n* = 23). * indicates statistical significance.

	*exoY*	*exoU*	*exoS*	*aprA*	*algD*	*plC N*	*pilA*	*pilB*
C6-HSL	0.8070	0.8070	0.9847	0.6193	0.2801	0.6129	0.0742 *	0.3467

## Data Availability

The original contributions presented in this study are included in the article/Supplementary Materials. Further inquiries can be directed to the corresponding authors.

## Supplementary Materials

The following supporting information can be downloaded at: https://www.mdpi.com/article/10.3390/pathogens14060536/s1, Table S1: Raw data of the testing results for the evaluated strains—*Pseudomonas aeruginosa* genes, quorum sensing assay, and quantitative biofilm evaluation of mono vs. mixed biofilm, Table S2: Raw data on the results for the evaluated strains (CFU/mL assay), Table S3: Raw data on the results for the evaluated strains (TTC assay), Table S4: Raw data on the results for the evaluated strains (CV assay), Table S5: Mixed biofilm groups

## Abbreviations

The following abbreviations are used in this manuscript:

CV	crystal violet assay
TTC	triphenyl tetrazolium chloride assay

## Appendix A

Monoculture and mixed chronic wound infections between 2016 and 2018 in University Hospital No. 1 in Bydgoszcz, Poland (*n* = 1142)—prevalence of bacterial species.

**Table A1:** Monoculture and mixed chronic wound infections between 2016 and 2018 in University Hospital No. 1 in Bydgoszcz, Poland (*n* = 1142).

Infections Prevalence by Number of Species Involved	Percentage
Monoculture (one species)	24.9
Mixed culture (two and more species)	75.1

**Table A2:** Prevalence of bacterial species in monoculture and mixed chronic wound infections between 2016 and 2018 in University Hospital No. 1 in Bydgoszcz, Poland (*n* = 1142).

Species	Subgroup	Percentage
*Staphylococcus aureus*		46.1
	MRSA	3.0
*Pseudomonas aeruginosa*		35.0
*Proteus mirabilis*		10.6
*Streptococcus agalactiae*		5.1
*S. pyogenes*		2.4
Other *		0.8

* Including *Streptococcus pyogenes*, *Streptococcus dysgalactiae*, *Proteus vulgaris*, *Proteus penneri*, *Enterobacter* spp., *Klebsiella* spp., *Serratia* spp., *Providencia* spp., *Citrobacter* spp., *Escherichia* spp., *Morganella* spp., *Acinetobacter* spp., and *Enterococcus* spp.; MRSA—methicillin-resistant *Staphylococcus aureus*.

**Table A3:** Prevalence of bacterial species in mixed chronic wound infections induced by two bacterial species (*n* = 154) between 2016 and 2018 in University Hospital No. 1 in Bydgoszcz, Poland.

Bacterial Species	Percentage
*Pseudomonas aeruginosa* and *Staphylococcus aureus*	29.2
*P. aeruginosa* and *Proteus mirabilis*	13.6
*S. aureus* and *Streptococcus agalactiae*	11.0
*S. aureus* and *Enterobacter cloacae*	10.4
*S. aureus* and *S. dysgalactiae*	9.7
*S. aureus* and *S. pyogenes*	9.7
*S. aureus* and *P. mirabilis*	7.8
*P. aeruginosa* and *S. dysgalactiae*	5.2
*P. aeruginosa* and *Serratia marcescens*	3.2
Other	0.2

## Appendix B

### Appendix B.1

The antimicrobial susceptibility tests of *Proteus mirabilis* and *Pseudomonas aeruginosa* strains were performed using BD Phoenix M50 (Becton-Dickinson, Franklin Lakes, NJ, USA) and BD Phoenix NMIC-402 panels (Becton-Dickinson, Franklin Lakes, NJ, USA). The interpretation was performed according to EUCAST recommendations [2].

**Table A4:** Antimicrobial susceptibility of the investigated strains, expressed as percentage of susceptible * strains (*Proteus mirabilis n* = 23, *Pseudomonas aeruginosa n* = 23).

Antimicrobial	Species	
	*Proteus mirabilis*	*Pseudomonas aeruginosa*
TZP	100.00	56.52
CAZ	95.65	60.87
FEP	82.61	60.87
IPM	100.00	0.00
MEM	100.00	56.52
AMK	95.65	86.96
NN	69.57	60.87
CIP	47.83	60.87
LEV	47.83	56.52
SXT	30.43	-

* Susceptible, increased exposure strains as defined by EUCAST [2] were included into the “susceptible” strains category; TZP—piperacillin/tazobactam, CAZ—ceftazidime, FEP—cefepime, IPM—imipenem, MEM—meropenem, AMK—amikacin, NN—tobramycin, CIP—ciprofloxacin, LEV—levofloxacin, SXT—trimethoprim/sulfamethoxazole, “-” expected resistant phenotype.

### Appendix B.2

The four distinctive groups of mixed biofilm pairs characterized by biofilm biomass (crystal violet assay, CV) and biofilm cell activity (triphenyl tetrazolium salt assay, TTC) were paired in cross-tabulation with other characteristics, such as wound characterization based on the underlying condition, *Pseudomonas aeruginosa* and *Proteus mirabilis* susceptibility patterns, and the presence of *Pseudomonas aeruginosa* virulence genes in the bacterial genome. To emphasize the differences between four groups, here, we briefly present the results of TTC and CV tests: group 1—TTC ↓, CV ↑ (*n* = 5), group 2—TTC ↑, CV ↓ (*n* = 5), group 3—TTC ↓, CV ↓ (*n* = 11), and group 4—TTC ↑, CV ↑ (*n* = 2). For more information, see Section 2.1 and Section 2.2.

**Table A5:** Characterization of wounds from which *Pseudomonas aeruginosa* isolates originate (*n* = 23). Five chronic wound groups were distinguished: arterial ulcers caused by venous insufficiency, diabetic foot ulcers, pressure injuries, and non-healing surgical wounds. For one isolate, the origin was not possible to determine; group 1—TTC ↓, CV ↑ (*n* = 5), group 2—TTC ↑, CV ↓ (*n* = 5), group 3—TTC ↓, CV ↓ (*n* = 11), and group 4—TTC ↑, CV ↑ (*n* = 2).

Wound Characterization	Group 1	Group 2	Group 3	Group 4
Arterial ulcers	1	2	2	0
Ulcers caused by venous insufficiency	0	0	1	0
Diabetic foot ulcers	1	1	3	0
Pressure injuries	3	1	4	1
Non-healing surgical wounds	0	1	0	1
Unknown origin	-	-	1	-

CV—crystal violet assay, TTC—triphenyl tetrazolium salt assay.

**Table A6:** Characterization of *Pseudomonas aeruginosa* isolate susceptibility * (*n* = 23); group 1—TTC ↓, CV ↑ (*n* = 5), group 2—TTC ↑, CV ↓ (*n* = 5), group 3—TTC ↓, CV ↓ (*n* = 11), and group 4—TTC ↑, CV ↑ (*n* = 2).

*Pseudomonas aeruginosa* Susceptibility	Group 1	Group 2	Group 3	Group 4
TZP	2	4	7	0
CAZ	3	4	7	0
FEP	3	4	7	0
IPM	0	0	0	0
MEM	2	3	7	1
AMK	3	3	8	2
NN	2	5	10	2
CIP	1	4	8	1
LEV	0	4	8	1

* Susceptible, increased exposure strains defined by EUCAST (2) were included in the “susceptible” category. TZP—piperacillin/tazobactam, CAZ—ceftazidime, FEP—cefepime, IPM—imipenem, MEM—meropenem, AMK—amikacin, NN—tobramycin, CIP—ciprofloxacin, LEV—levofloxacin, CV—crystal violet assay, TTC—triphenyl tetrazolium salt assay.

**Table A7:** Characteristics of *Proteus mirabilis* isolate susceptibility * (*n* = 23); group 1—TTC ↓, CV ↑ (*n* = 5), group 2—TTC ↑, CV ↓ (*n* = 5), group 3—TTC ↓, CV ↓ (*n* = 11), and group 4—TTC ↑, CV ↑ (*n* = 2).

*Proteus mirabilis* Susceptibility *	Group 1	Group 2	Group 3	Group 4
TZP	5	5	11	2
CAZ	5	4	11	2
FEP	4	4	9	2
IPM	5	5	11	2
MEM	5	5	11	2
AMK	5	4	11	2
NN	3	3	8	2
CIP	1	4	6	0
LEV	1	4	6	0
SXT	0	2	5	1

* Susceptible, increased exposure strains as defined by EUCAST [2] were included into the “susceptible” category. TZP—piperacillin/tazobactam, CAZ—ceftazidime, FEP—cefepime, IPM—imipenem, MEM—meropenem, AMK—amikacin, NN—tobramycin, CIP—ciprofloxacin, LEV—levofloxacin, SXT—trimethoprim/sulfamethoxazole, CV—crystal violet assay, TTC—triphenyl tetrazolium salt assay.

**Table A8:** Presence of *Pseudomonas aeruginosa* virulence genes in isolates (*n* = 23); group 1—TTC ↓, CV ↑ (*n* = 5), group 2—TTC ↑, CV ↓ (*n* = 5), group 3—TTC ↓, CV ↓ (*n* = 11), and group 4—TTC ↑, CV ↑ (*n* = 2).

Gene	Group 1	Group 2	Group 3	Group 4
*toxA*	5	5	11	2
*lasB*	5	5	11	2
*exoT*	5	5	11	2
*exoY*	5	3	11	1
*exoU*	4	3	11	2
*exoS*	1	4	3	0
*aprA*	3	3	8	2
*algD*	3	2	6	1
*plC H*	5	5	11	2
*plC N*	2	2	5	0
*pilA*	2	1	3	2
*pilB*	1	0	1	0

CV—crystal violet assay, TTC—triphenyl tetrazolium salt assay.
